# Luteolin Potentially Treating Prostate Cancer and COVID-19 Analyzed by the Bioinformatics Approach: Clinical Findings and Drug Targets

**DOI:** 10.3389/fendo.2021.802447

**Published:** 2022-02-01

**Authors:** Yu Ye, Ziyan Huang, Manying Chen, Yongfeng Mo, Zengnan Mo

**Affiliations:** ^1^ Department of Urology, The First Affiliated Hospital of Guangxi Medical University, Nanning, China; ^2^ Health Management Department, First Affiliated Hospital of Guangxi Medical University, Nanning, China; ^3^ Center for Genomic and Personalized Medicine, Guangxi Medical University, Nanning, China; ^4^ Department of Emergency Medicine, The Second Affiliated Hospital of Guangxi Medical University, Nanning, China

**Keywords:** prostate cancer, COVID-19, clinical feature, luteolin, drug target, mechanism

## Abstract

Coronavirus disease 2019 (COVID-19) is a serious epidemic, characterized by potential mutation and can bring about poor vaccine efficiency. It is evidenced that patients with malignancies, including prostate cancer (PC), may be highly vulnerable to the SARS-CoV-2 infection. Currently, there are no existing drugs that can cure PC and COVID-19. Luteolin can potentially be employed for COVID-19 treatment and serve as a potent anticancer agent. Our present study was conducted to discover the possible drug target and curative mechanism of luteolin to serve as treatment for PC and COVID-19. The differential gene expression of PC cases was determined *via* RNA sequencing. The application of network pharmacology and molecular docking aimed to exhibit the drug targets and pharmacological mechanisms of luteolin. In this study, we found the top 20 up- and downregulated gene expressions in PC patients. Enrichment data demonstrated anti-inflammatory effects, where improvement of metabolism and enhancement of immunity were the main functions and mechanism of luteolin in treating PC and COVID-19, characterized by associated signaling pathways. Additional core drug targets, including *MPO* and *FOS* genes, were computationally identified accordingly. In conclusion, luteolin may be a promising treatment for PC and COVID-19 based on bioinformatics findings, prior to future clinical validation and application.

## Introduction

Epidemiological findings suggest that coronavirus disease 2019 (COVID-19) has evolved and mutated around the world, leading to higher fatality rates (1). Mutations generating new variants may endow the virus with potent survival evolution causing refractory characteristics (2). There may be extensive vaccinations worldwide but mutated severe acute respiratory syndrome coronavirus 2 (SARS-CoV-2) affects the protective efficacy of these vaccines (3). Therefore, it is believed that exploring effective bioactive agents for treating COVID-19 is an imperative task. Other existing data show that SARS-CoV-2-infected tumor cases may induce increased lethality rate than tumor-free patients based on prospective cohort analysis (4). Owing to COVID-19 potentially impacting cancer patients, an effective strategy is critically warranted. Prostate cancer (PC) is cancer of the prostate gland and is known for its potent invasiveness and heterogeneous metastasis (5). Based on epidemiological investigation assays, PC is one of most common malignant tumors in men around the world (6). Since SARS-CoV-2 has been prevalent, hospitalization is optimized for those with coronavirus infection, which then disseminates to other patients, including PC cases (7). As result, inpatients with PC may undertake greater risk in novel coronavirus exposure and infection. It is visibly reasoned that PC patients with COVID-19 can be refractory to commonly used treatment as effective clinical prescription is non-existent. Therefore, we need to screen and explore some candidate bioactive ingredients to treat PC and COVID-19, which poses as a current challenge.

Luteolin, a naturally forming flavonoid, is well-evidenced with beneficial functions, such as anticancer (8), neuroprotection (9), and hepatoprotection (10). As a potential anticancer agent, luteolin is examined preclinically with anti-PC functions through regulating Wnt signaling (11), Anoctamin 1 activity (12), and miR-301 expression (13). Interestingly, other *in vitro* data suggest that luteolin restrains viral-caused inflammatory stress (14). Nevertheless, the curative benefits and mechanism of luteolin in treating PC and COVID-19 remain uninvestigated currently. It is widely reported that a bioinformatics methodology using network pharmacology and/or molecular docking can be applied for deciphering the drug targets and therapeutic mechanisms in bioactive agents in the treatment of complicated diseases, such as hepatocholangiocarcinoma (15), leukemia (16), and cleft lip (17). Here, we aimed to use both network pharmacology and molecular docking approaches to screen and reveal all drug targets, pharmacological functions, and curative mechanisms of luteolin against PC and COVID-19 before any clinical trial.

## Materials and Methods

### Identification of Prostate Cancer/COVID-19-Associated Genes

The TCGA-RNA sequences of PC patients were downloaded from the UCSC Xena platform, wherein the “limma” package of R language was used, with a false discovery rate <0.05 and |log^fold change (FC)^| >1 to identify the differential expression genes. The top 20 significantly upregulated and downregulated genes were obtained for drawing the differential gene volcano map. The associated genes of COVID-19 were selected from the GeneCards database, Online Mendelian Inheritance in Man (OMIM) database, and NCBI gene function module. Finally, these genes were compared to obtain the overlapping targets in prostate cancer and COVID-19 through the “Venn diagram” package of R language (18, 19).

### Detection of the Targets in Luteolin

The database-based tools of Traditional Chinese Medicine Systems Pharmacology Database and Analysis Platform (TCMSP), Comparative Toxicogenomics Database (CTD), Swiss Target Prediction, and PharmMapper (8) were used to detect the candidate genes of luteolin (15). The resulting data were revised using the Swiss-Prot database (reviewed) and human settings in the UniProt database (20).

### Enrichment Analyses and Network Visualization

R-language packages, including “ClusterProfiler” and “GOplot”, were used for the enrichment analysis and visualization of the Gene Ontology (GO), biological process (BP), and KEGG pathways of the overlapping gene targets of luteolin in PC/COVID-19. The cutoff value for both the *p*- and *q*-values was set to 0.05 for enriching and plotting the bubble chart, bar chart, and circle chart (21). Cytoscape_v3.8.2 was used to construct a drug–target–GO function–pathway–disease for creating GO and pathway in response to targeting luteolin against PC and COVID-19 intersecting genes (22).

### Plotting Protein–Protein Interaction Network and Discovering Core Targets

The target of luteolin for the treatment of PC and COVID-19 was obtained after mapping *via* the STRING database to obtain the network interaction relationship between the target–target function-related proteins and the target protein–protein interaction (PPI) network diagram (23). Using the Network Analyzer in Cytoscape_v3.8.2 to analyze topological parameters such as the median and maximum degrees of freedom in the network, all core targets were identified according to the degree value. The upper limit of the filtering range was the maximum degree value in the topology data, and the lower limit was the median degree of freedom number (24).

### Molecular Docking Analysis

According to the degree value parameters, myeloperoxidase (MPO) and FOS core targets were selected for molecular docking. The structure of luteolin was obtained from the PubChem database, and the protein structure associated with core genes was acquired from the PDB database. Using the ChemBio3D Draw module in the ChemBioOffice 2010 software, three-dimensional structures of these compounds were optimized for the molecular force field 2 (MM2). The Autodock Tools 1.5.6 tool of Autodock software was used to process the corresponding proteins, including hydrogenate, Gasteiger charge, and merge non-polar hydrogen. The original pdb file format was converted into the pdbqt file format recognized by the Autodock Vina program, performing a ligand basis for molecular docking. Based on the root mean square deviation (RMSD) between the docked and original ligand molecule, the rationality of the docking parameter settings could be assessed. It was generally concluded that RMSD ≤4 Å was the threshold for the conformation of the ligand to match the conformation of the original ligand after docking (25, 26).

## Results

### Identification of PC/COVID-19-Associated Genes

**Figure 1:**
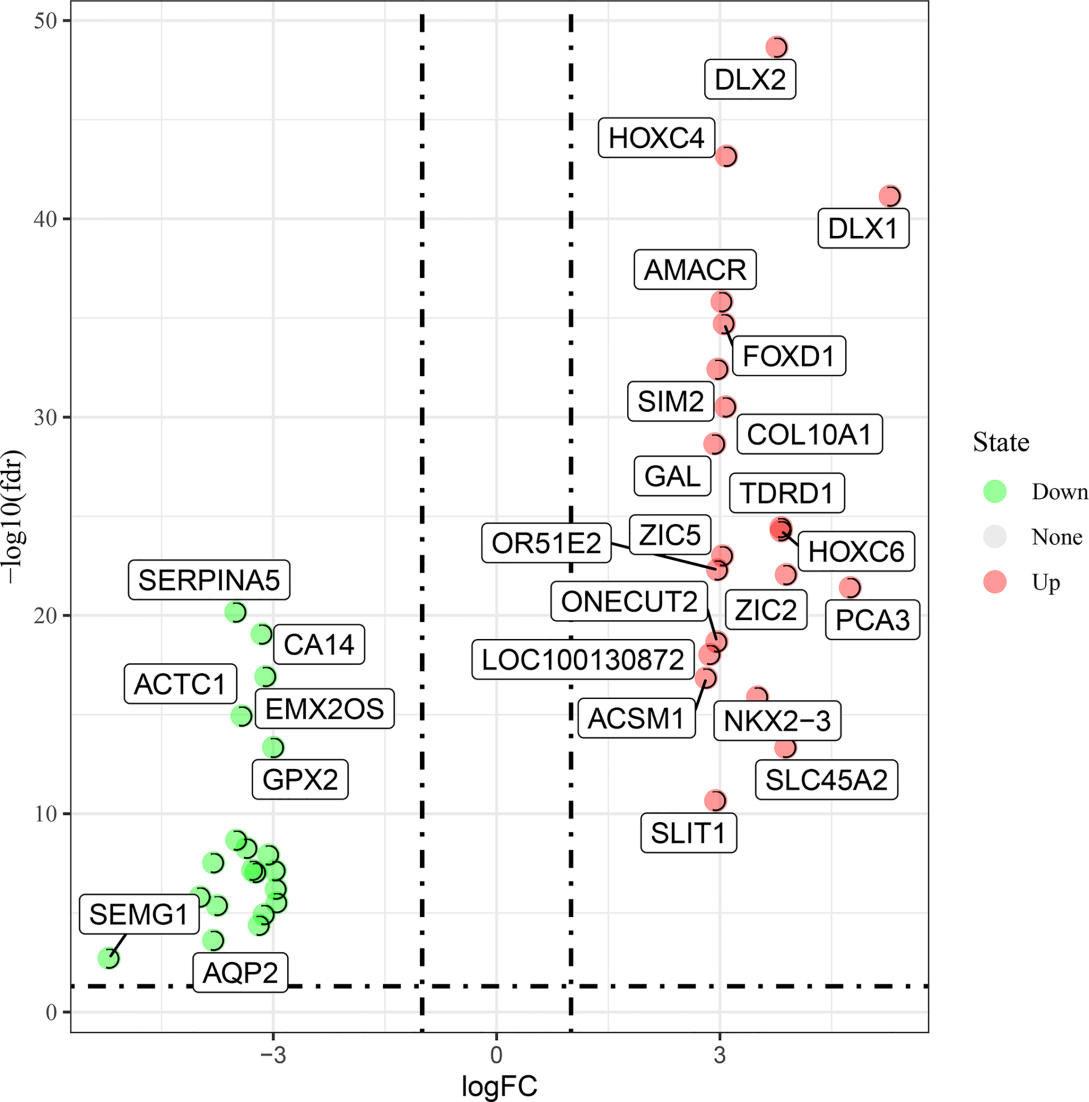
Top up- and downregulated expression genes of prostate cancer (PC) cases were characterized in the volcano map.

**Figure 2:**
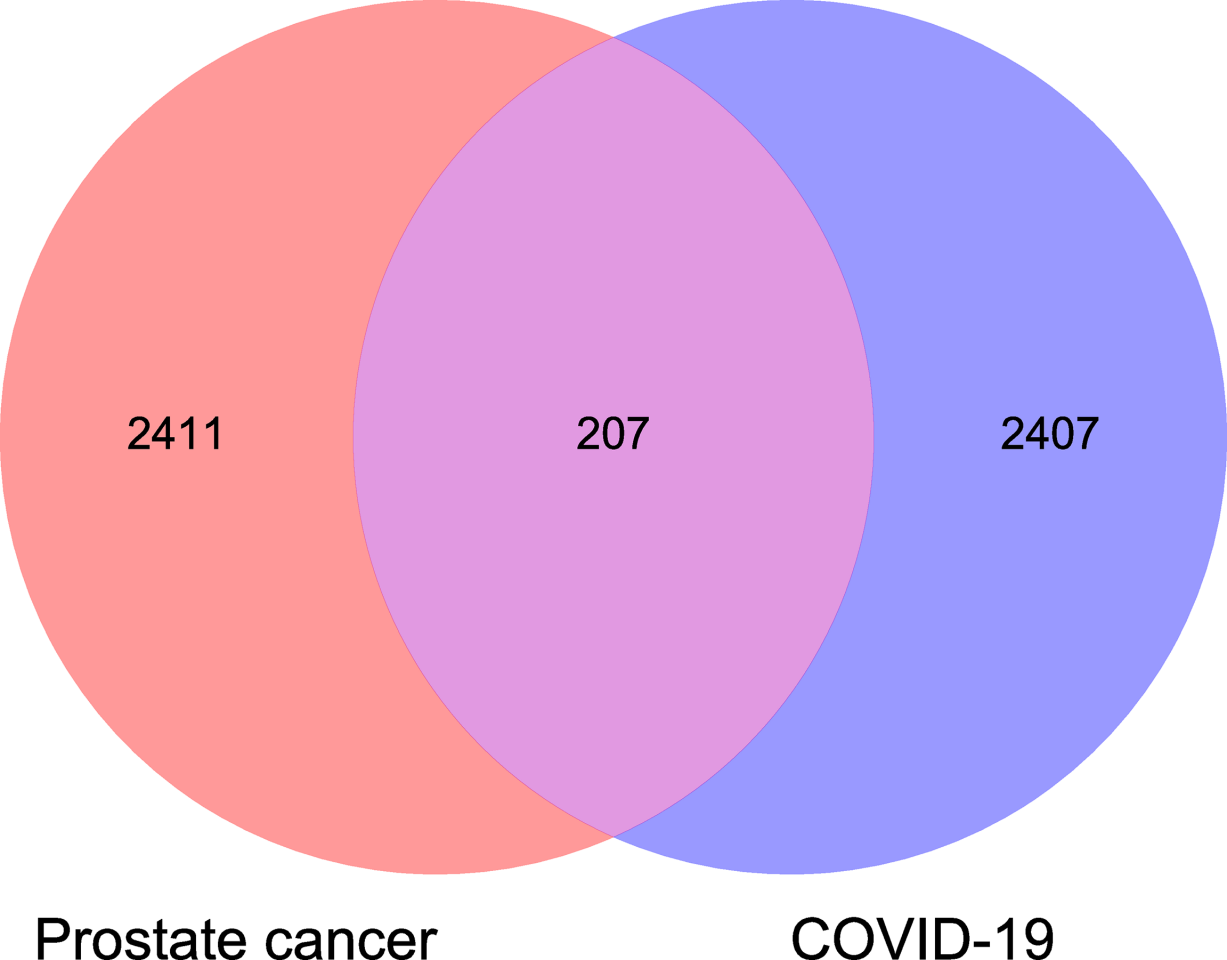
Venn diagram analysis showed all common and shared target genes in PC and coronavirus disease 2019 (COVID-19).

TCGA-RNA sequencing analysis showed that a total of 550 samples, consisting of 52 normal samples and 498 tumor samples, were obtained for comparative determination. The data included 2,618 significantly differentially expressed genes in PC. Furthermore, the top 20 distinctly upregulated and downregulated genes were used to display the differential gene volcano map (**Figure 1**). In addition, 2,614 targets of COVID-19 were identified. The Venn diagram between PC targets and COVID-19-related targets is shown in **Figure 2**, wherein 207 intersection targets in PC and COVID-19 were acquired.

### Detection of the Targets in Luteolin and PC/COVID-19

**Figure 3:**
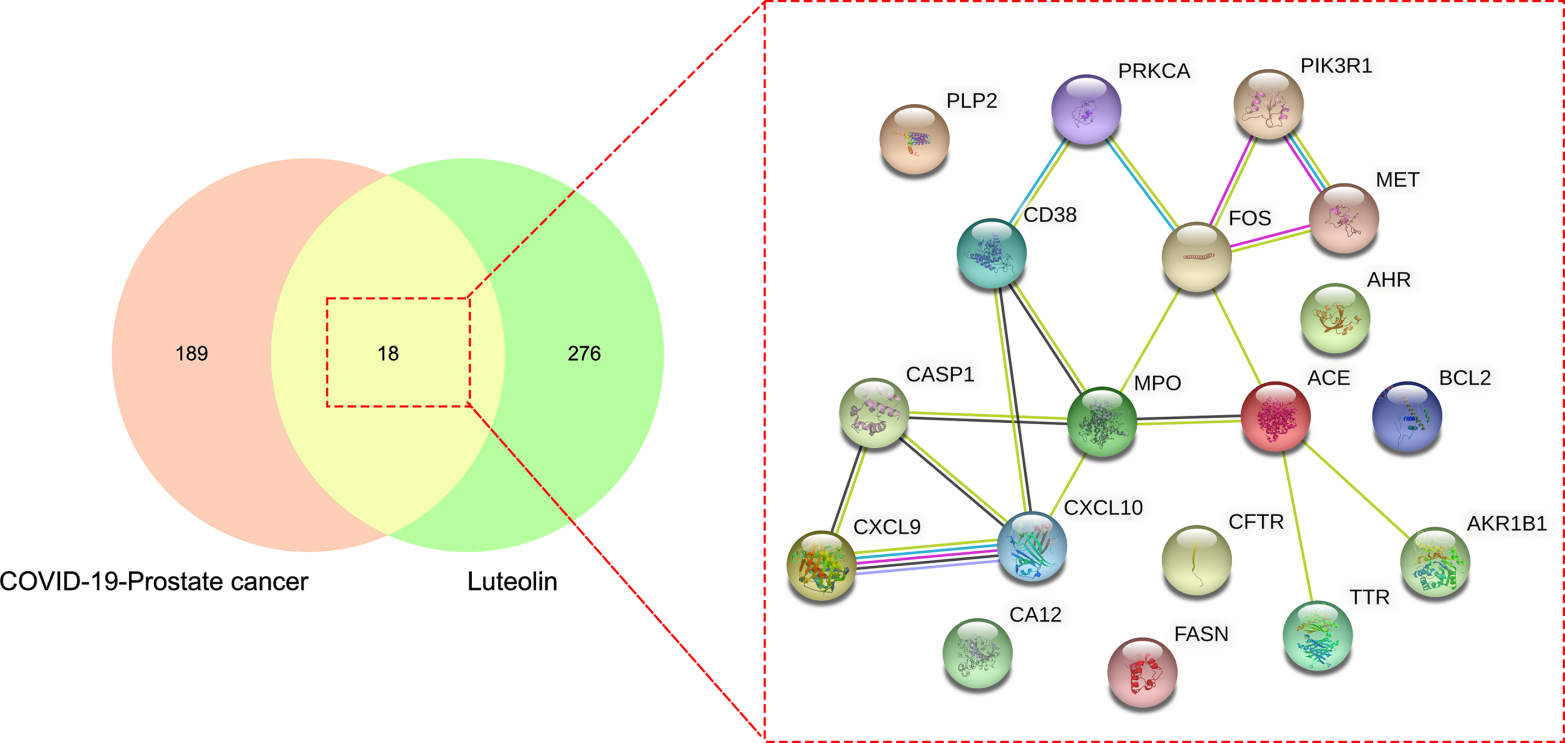
Further Venn diagram assay showed other common and shared target genes of luteolin in treating PC and COVID-19.

The drug targets of luteolin were searched through the TCMSP database, CTD, and other databases, and 294 targets of luteolin were ascertained after the removal of duplicates and correction by the UniProt database. Finally, the drug target and disease intersection target were mapped and uploaded to the Venn diagram analysis, and a total of 18 intersection targets were obtained (**Figure 3**).

### Enrichment Exhibition and Network Visualization

**Figure 4:**
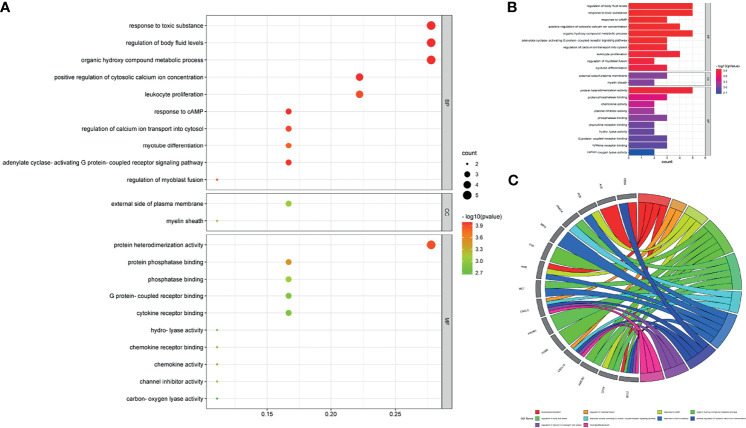
Enrichment annotation exhibited GO data of luteolin in treating PC and COVID-19, as characterized in the bubble graph **(A)**, histogram graph **(B)**, and circle chart **(C)**.

**Figure 5:**
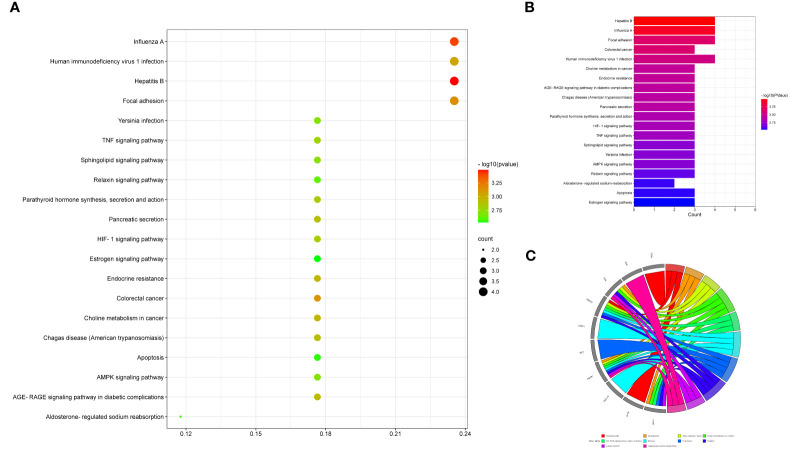
Enrichment analysis exhibited KEGG data of luteolin in treating PC and COVID-19, as characterized in the bubble graph **(A)**, histogram graph **(B)**, and circle chart **(C)**.

After performing GO and KEGG pathway enrichment analyses using intersection targets through R language-related packages, output data were summarized and exhibited in the GO-linked bubble chart (**Figure 4A**), histogram (**Figure 4B**), and circle chart (**Figure 4C**) and KEGG pathway enrichment-displayed bubble chart (**Figure 5A**), bar chart (**Figure 5B**), and circle chart (**Figure 5C**). The results show that the BPs of drug genes were mainly involved in the response to toxic substances, regulation of body fluid levels, response to cAMP, positive regulation of cytosolic calcium ion concentration, organic hydroxy compound metabolic process, adenylate cyclase-activating G protein-coupled receptor signaling pathway, regulation of calcium ion transport into cytosol, leukocyte proliferation, regulation of myoblast fusion, and myotube differentiation. The cellular component (CC) of drug genes mainly included the external side of the plasma membrane and myelin sheath. The molecular function (MF) was widely comprised of protein heterodimerization activity, protein phosphatase binding, chemokine activity, channel inhibitor activity, phosphatase binding, chemokine receptor binding, hydro-lyase activity, G protein-coupled receptor binding, cytokine receptor binding, and carbon–oxygen lyase activity.

**Figure 6:**
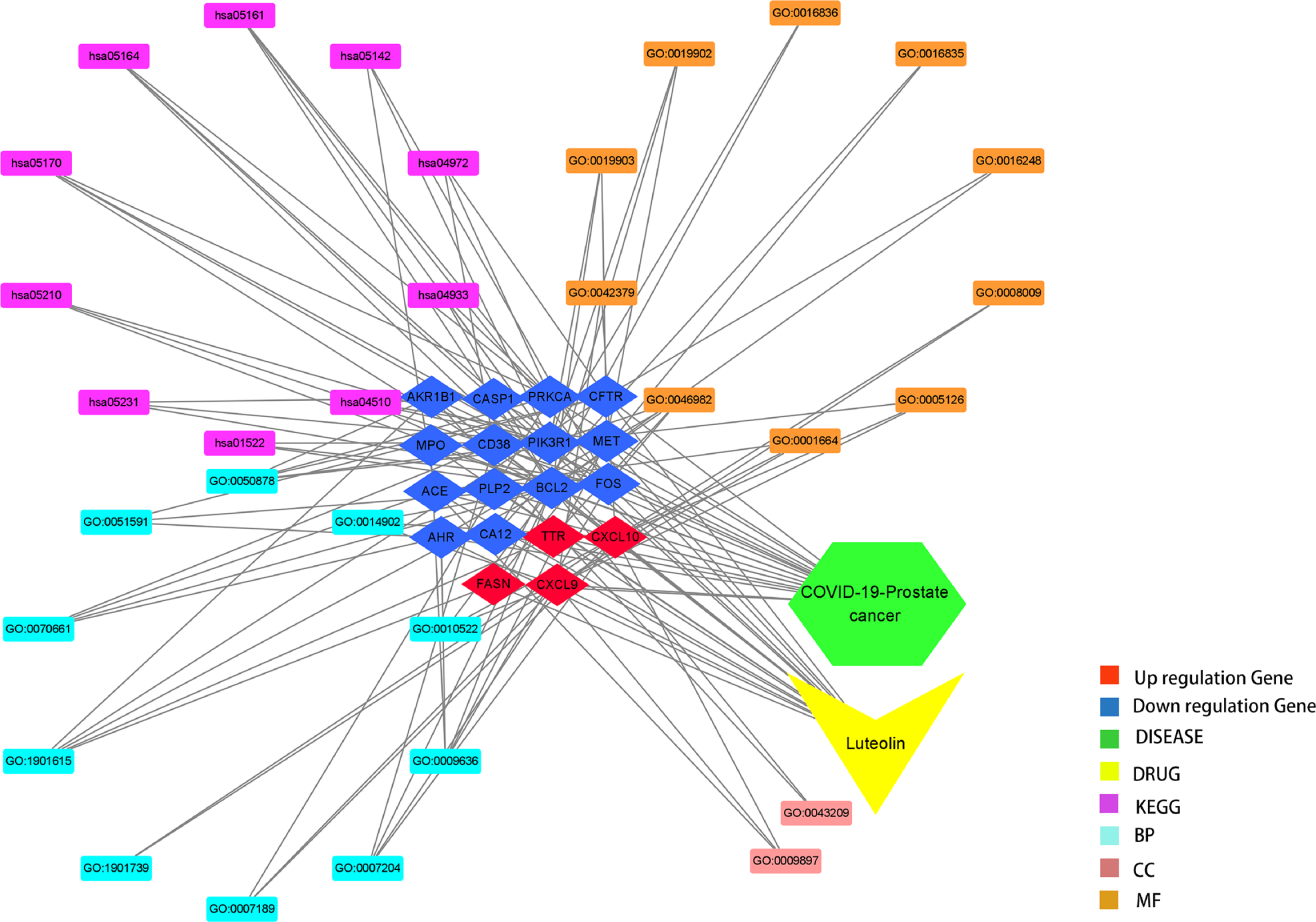
Composite graph of luteolin targeting genes–enrichment pathways–PC/COVID-19.

It is found that a total of 73 KEGG pathways (*P* < 0.05) were chiefly involved in hepatitis B, influenza A, focal adhesions, colorectal cancer, human immunodeficiency virus 1 infection, endocrine resistance, choline metabolism in cancer, AGE-RAGE signaling pathway in diabetic complications, pancreatic secretions, Chagas disease (American trypanosomiasis), parathyroid hormone synthesis, secretion and action, HIF-1 signaling pathway, TNF signaling pathway, sphingolipid signaling pathway, AMPK signaling pathway, *Yersinia* infection, relaxin signaling pathway, aldosterone-regulated sodium reabsorption, apoptosis, and estrogen signaling pathway. In addition, drug targeting genes–GO findings, including the BP, CC, and MF-pathway–disease, were exhibited in the visualization graph (**Figure 6**).

### Plotting Interaction Network and Discovering Core Targets

**Figure 7:**
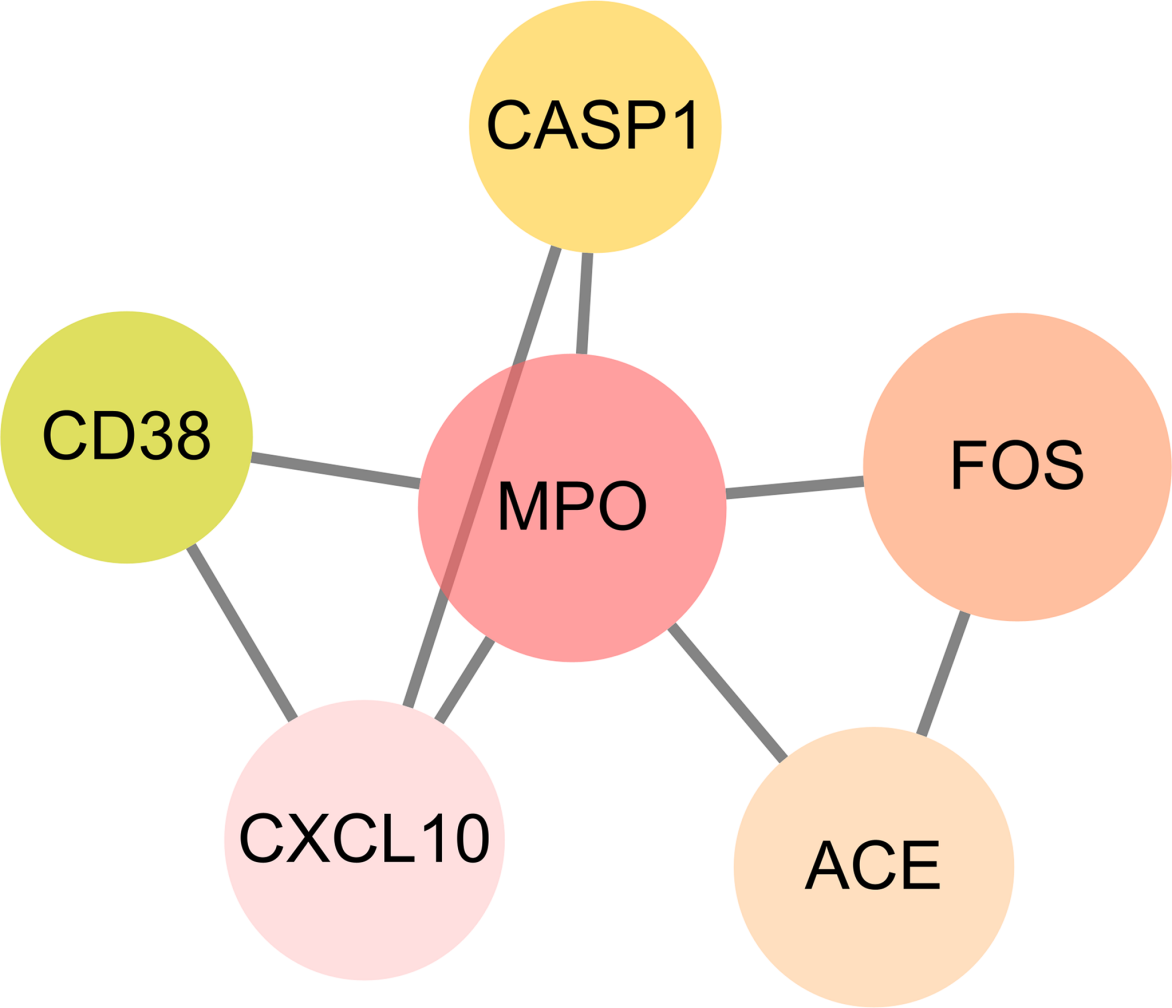
Identification of all core drug targets in luteolin in treating PC and COVID-19.

The intersection targets were imported into the Cytoscape_v3.8.2 to calculate topological parameters of luteolin in treating PC and COVID-19 for the PPI network. The median degree of target freedom was 3, while the maximum degree of freedom was 5. All six core drug targets were ascertained, namely, MPO, FOS, ACE, CXCL10, CASP1, and CD38 (**Figure 7**).

### Molecular Docking Discovery

**Figure 8:**
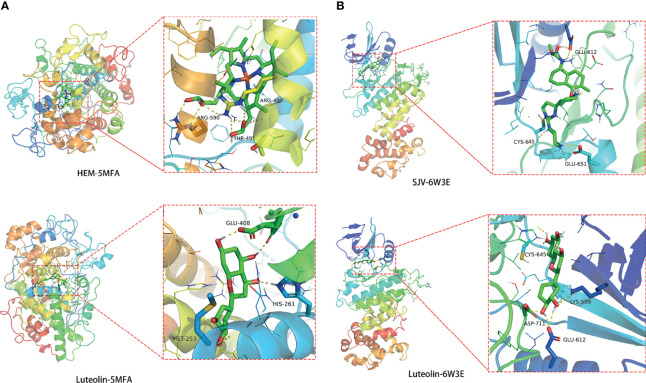
Molecular docking findings of luteolin in treating PC and COVID-19, as revealed in MPO-5MFA **(A)** and FOS-6W3E **(B)**.

The MPO and FOS core proteins we selected and docked with 5MFA and 6W3E, respectively. In the 5MFA in MPO protein, the enaction parameters with the active cavity box model were *x* = 40, *y* = 40, *z* = 40, and spacing = 0.375, and center-*x*, *y*, and *z* were −21.662, 0.588, and 5.123, respectively. The RMSD in the original ligand was 1.70 Å. The hydrogen bond between the original ligand HEM and 5MFA protein acted on the amino acid residues, including ARG-499, ARG-590, and THR-495. The free docking energy with the protein was −10.81 kcal/mol. Luteolin-shaped hydrogen bonds with amino acid residues, including GLU-408, MET-253, and HIS-261. The free docking energy with protein was −7.65 kcal/mol, indicating potent binding activity with the 5MFA protein (**Figure 8A**).

In the 6W3E in the FOS protein, the enaction parameters with the active cavity box model were *x* = 40, *y* = 40, *z* = 40, and spacing = 0.375, and center-*x*, *y*, and *z* were 0.248, −43.209, and 45.793, respectively. The RMSD in the original ligand was 2.72 Å. The hydrogen bond between the original ligand SJV and 6W3E protein acted on the amino acid residues, including GLU-612, CYS-645, and GLU-651. The free docking energy with the protein was −9.01 kcal/mol. Luteolin-shaped hydrogen bonds with amino acid residues, including CYS-645, LYS-599, ASP-711, and GLU-612. The free docking energy with protein was −6.89 kcal/mol, indicating potent binding activity with the 6W3E protein (**Figure 8B**).

## Discussion

The COVID-19 pandemic brought by SARS-CoV-2 has caused a tremendous death toll and economic burden in the world, killing at least three million infected persons (27). In the current existing clinical treatment, some chemotherapeutics that may be used in the clinics, such as hydroxychloroquine, dexamethasone, and lopinavir–ritonavir, may be prescribed for potential COVID-19 therapy. However, the drug response, interaction, or side effect may be undefined (28). Encouragingly, parts of the medical vaccine have been used for immunization against COVID-19 in humans, including mRNA vaccines (29). Statistical analysis data indicate that non-infectious conditions, such as diabetes and cancers, have numerous cases worldwide (30). Among cancer cases, the tumor microenvironment may be altered *via* depositing metabolites as a result of reduced immunity (31), possibly causing increased risk of infections including SARS-CoV-2 (32). It is presently reported that the COVID-19 pandemic and uncertain SARS-CoV-2 variants are still persisting and evolving diffusely. Epidemiologically, low- or middle-income countries display rising mortality caused by COVID-19, such as in India and Pakistan (33). In the recent decade, the incidence and mortality of PC in Asians have shown an elevated trend, leading to a global health challenge (34). In China, the incidence of prostate cancer among Chinese men is rising rapidly due to the increasing aging population (35). In addition, the immunosuppressed PC patients may have a higher risk of SARS-CoV-2 infection during COVID-19 outbreaks. The current prescribed medicines for PC and COVID-19 therapy are limited as SARS-CoV-2 mutations pose an additional challenge.

As a pharmacologically bioactive compound, luteolin may potentially serve as treatment for PC and COVID-19. By using bioinformatics determination, all 207 shared genes in PC and COVID-19 were confirmed accordingly. The TCGA-RNA seq assay displayed all the top 20 up- and downregulated genes in PC patients. The difference analysis may be used to clinically feature PC and COVID-19 cases. Collectively, 207 intersection genes of PC and COVID-19 were identified as potential pharmacological targets. Based on the network pharmacology analysis, other 18 intersection genes and six core genes in luteolin against PC and COVID-19 were determined prior to further identification with molecular docking. The computed data suggested that these *MPO* and *FOS* genes may be potential drug targets of luteolin action in treating PC and COVID-19. MPO, a leukocyte enzyme, has various functions such as mediating inflammatory response and immunoregulation, and it can participate in the occurrence and development of diseases (36). It is found that MPO may be a potential target for prostatic cancer and COVID-19 (37, 38). FOS can be activated *via* a wide range of stimuli and is an early transcription marker for neural activity (39). Early reports show that *Fos* may be a proapoptotic gene in prostate cancer (40). Analyzed by enrichment assay, these bioinformatics data exhibited that luteolin potently treated PC and COVID-19 through anti-inflammatory effects, improving metabolism and enhancing immunity, characterized by associated signaling pathways. Taken together, luteolin-mediated anti-PC and COVID-19 benefits could be accomplished through regulation of core gene activities, especially MPO and FOS. As seen in the results, it is possible that luteolin monotherapy or combined therapy may be used for clinical PC and COVID-19 treatment.

## Conclusions

Our bioinformatics data reveal the drug targets and pharmacological mechanisms of luteolin, characterized by enrichment findings. In future studies, luteolin may be potentially applied for the clinical treatment of PC and COVID-19 after experimental validation.

## Data Availability Statement

The original contributions presented in the study are included in the article/**Supplementary Material**. Further inquiries can be directed to the corresponding author.

## Author Contributions

YY and ZM conceived and designed the study. ZH, MC, and YM performed the data analysis and data interpretation. YY and ZH conducted the bioinformatics and statistical analyses. YY and ZM prepared the manuscript. All authors contributed to the article and approved the submitted version.

## Funding

This study was funded by the National Key Research and Development Program of China (No. 2017YFC0908000), National Natural Science Foundation of China (No. 81770759), Major Project of Guangxi Innovation Driven (No. AA18118016), and Guangxi Key Laboratory for Genomic and Personalized Medicine (Nos. 16-380-54, 17-259-45, 19-050-22, 19-185-33, 20-065-33).

## Conflict of Interest

The authors declare that the research was conducted in the absence of any commercial or financial relationships that could be construed as a potential conflict of interest.

## Publisher’s Note

All claims expressed in this article are solely those of the authors and do not necessarily represent those of their affiliated organizations, or those of the publisher, the editors and the reviewers. Any product that may be evaluated in this article, or claim that may be made by its manufacturer, is not guaranteed or endorsed by the publisher.
